# Assessing the asymptomatic proportion of SARS-CoV-2 infection with age in China before mass vaccination

**DOI:** 10.1098/rsif.2022.0498

**Published:** 2022-10-12

**Authors:** Zengmiao Wang, Peiyi Wu, Jingyuan Wang, José Lourenço, Bingying Li, Benjamin Rader, Marko Laine, Hui Miao, Ligui Wang, Hongbin Song, Nita Bharti, John S. Brownstein, Ottar N. Bjornstad, Christopher Dye, Huaiyu Tian

**Affiliations:** ^1^ State Key Laboratory of Remote Sensing Science, Center for Global Change and Public Health, College of Global Change and Earth System Science, Beijing Normal University, Beijing, People's Republic of China; ^2^ School of Computer Science and Engineering, Beihang University, Beijing, People's Republic of China; ^3^ Peng Cheng Laboratory, Shenzhen, People's Republic of China; ^4^ Biosystems and Integrative Sciences Institute, University of Lisbon, Lisbon, Portugal; ^5^ Computational Epidemiology Lab, Boston Children's Hospital, Boston, MA, USA; ^6^ Department of Epidemiology, Boston University School of Public Health, Boston, MA, USA; ^7^ Meteorological Research Unit, Finnish Meteorological Institute, Helsinki, Finland; ^8^ Department of Statistics, College of Art and Science, Ohio State University, Columbus, OH, USA; ^9^ Center of Disease Control and Prevention, PLA, Beijing, People's Republic of China; ^10^ Center for Infectious Disease Dynamics, Department of Biology, Pennsylvania State University, University Park, PA, USA; ^11^ Department of Entomology, College of Agricultural Sciences, Pennsylvania State University, University Park, PA, USA; ^12^ Harvard Medical School, Harvard University, Boston, MA, USA; ^13^ Department of Biology, University of Oxford, Oxford, UK

**Keywords:** SARS-CoV-2, asymptomatic infection, age-stratified compartment model

## Abstract

Some asymptomatic individuals carrying SARS-CoV-2 can transmit the virus and contribute to outbreaks of COVID-19. Here, we use detailed surveillance data gathered during COVID-19 resurgences in six cities of China at the beginning of 2021 to investigate the relationship between asymptomatic proportion and age. Epidemiological data obtained before mass vaccination provide valuable insights into the nature of pathogenicity of SARS-CoV-2. The data were collected by multiple rounds of city-wide PCR testing with contact tracing, where each patient was monitored for symptoms through the whole course of infection. The clinical endpoint (asymptomatic or symptomatic) for each patient was recorded (the pre-symptomatic patients were classified as symptomatic). We find that the proportion of infections that are asymptomatic declines with age (coefficient = −0.006, 95% CI: −0.008 to −0.003, *p* < 0.01), falling from 42% (95% CI: 6–78%) in age group 0–9 years to 11% (95% CI: 0–25%) in age group greater than 60 years. Using an age-stratified compartment model, we show that this age-dependent asymptomatic pattern, together with the distribution of cases by age, can explain most of the reported variation in asymptomatic proportions among cities. Our analysis suggests that SARS-CoV-2 surveillance strategies should take account of the variation in asymptomatic proportion with age.

## 1. Introduction

The proportion of asymptomatic infections shows vast differences across the world, varying from 1.21% to 91.88% [1–3]. Once age-stratified, the fraction of asymptomatic cases also show substantial variation in age [4–6]. Due to the potential threat of asymptomatic transmission in the community [7–10], numerous studies have been performed to decipher the underlying mechanism [11–21] and quantify the major drivers of heterogenicity in asymptomatic infection [22,23].

The main obstacles to understanding the drivers of asymptomatic transmission include assessment of asymptomatic status (for example, some studies define asymptomatic cases only based on the symptom status when testing [24,25]), testing bias [23,26] and sampling bias (for example, testing is more likely to be completed in hospitalized individuals [27,28]). Because passive surveillance is often limited to symptomatic cases, a substantial proportion of asymptomatic cases are expected to be underreported [29,30]. Asymptomatic transmission poses a great challenge to local governments and health systems in terms of control policy [22,29,31–35]. Therefore, it is necessary to identify the factors driving such heterogeneity in asymptomatic proportions to design more efficiently surveillance measures that include such subclass of the infected.

To address this heterogenicity, intensive, active surveillance of SARS-CoV-2 infection is needed. Detailed surveillance data collected during COVID-19 resurgences in six cities of China in 2021 provide a valuable opportunity to study this issue. Multiple rounds of population-level PCR testing in each city accelerated the speed of SARS-CoV-2 infection identification (the cases have a short time interval between infection and detection). Besides, it also improved the detection rate among the currently infected irrespective of clinical outcome. The clinical endpoint (asymptomatic or symptomatic) for each patient was obtained by detailed surveillance across the whole course of infection. The pre-symptomatic individuals are classified as symptomatic because they will develop symptoms during the course of infection. More importantly, epidemiological data prior to mass vaccination provide valuable insight for the nature of pathogenicity of SARS-CoV-2. Using this unique dataset, we explored the potential factors that may contribute to observed variations in the proportion of asymptomatic infections, and reported thereof. Our analysis has implications for understanding the role of asymptomatic individuals in COVID-19 transmission and can help guide future disease control policies that depend on the frequency of asymptomatic cases as a hidden subgroup of infections.

## 2. Methods

### 2.1. The age-dependent asymptomatic proportion and the total asymptomatic proportion

Based on the observed data, the asymptomatic proportion is dependent on age. Let *p*_1_, *…*, *p*_7_ represent the asymptomatic proportion of detected cases in age groups 0–9, 10–19, 20–29, 30–39, 40–49, 50–59, 60+, respectively. So, the total asymptomatic proportion (*p*) is calculated as follows:

$$p=\frac{\sum_{i=1}^{G}p_{i}N_{i}}{\sum_{i=1}^{G}N_{i}},\;\text{where }N_{i}\;\text{ is the number of cases in age group }i.$$

The total asymptomatic proportion is a combination of the age-dependent asymptomatic proportion and the SARS-CoV-2 case distribution in age.

### 2.2. Age-stratified compartment model fit to detected SARS-CoV-2 data

Given the stochasticity and heterogeneity in the transmission of SARS-CoV-2 [36,37], we developed an age-stratified discrete stochastic compartment model that incorporated asymptomatic and symptomatic cases. Specifically, we consider susceptible (*S_i_*), latent (*L_i_*), pre-symptomatic (*L*_P*i*_), infectious asymptomatic (*I*_A*i*_), infectious symptomatic (*I*_S*i*_), removed symptomatic (*R*_S*i*_) and removed asymptomatic (*R*_A*i*_) individuals for age group *i*, *i* = 1,*…*,*G.* The latent compartment represents the individuals who are exposed to the virus but not contagious. The pre-symptomatic compartment represents the individuals who spread the virus before they develop symptoms, which is a feature of SARS-CoV-2. Note that control measures (population-level PCR tests and contact tracing) were implemented, so infected individuals will be identified through population-level testing and contact tracing. Therefore, they will be quarantined and exit the transmission chain. Isolation is the dominant factor in removed status. We assume that a constant proportion (θ) of infected people exit from compartments *L_i_*, *L*_P*i*_, *I*_A*i*_ and *I*_S*i*_. Individuals receiving PCR tests will stay in the *S* compartment if the test results are negative. Individuals exiting from *L_i_* compartment may develop symptoms with proportion of 1 − *p_i_*, where *p_i_* is the proportion of *L_i_* that becomes *I*_A*i*_ in age group *i*. We did not consider the Ct value from the PCR tests and assumed that the PCR tests will be positive if the individual is infected. The model structure and the parameters involved are shown in electronic supplementary material, figure S1. The full set of equations representing the transmission for age group *i* is given by

$$\begin{array}{c} S_{i}\left(t+1\right)=S_{i}\left(t\right)-\beta \mu_{i}S_{i}\left(t\right)\overset{G}{\underset{j=1}{\sum }}\frac{C_{ij}\left\{I_{Sj}\left(t\right)+rI_{Aj}\left(t\right)+kL_{Pj}\left(t\right)\right\}}{N_{j}}\\ L_{i}\left(t+1\right)=L_{i}\left(t\right)+\beta \mu_{i}S_{i}\left(t\right)\overset{G}{\underset{j=1}{\sum }}\frac{C_{ij}\left\{I_{Sj}\left(t\right)+rI_{Aj}\left(t\right)+kL_{Pj}\left(t\right)\right\}}{N_{j}}\\ -\theta L_{i}\left(t\right)-\sigma \left(1-\theta \right)p_{i}L_{i}\left(t\right)-\sigma \left(1-\theta \right)\left(1-p_{i}\right)L_{i}\left(t\right)\\ I_{Ai}\left(t+1\right)=I_{Ai}\left(t\right)+\sigma \left(1-\theta \right)p_{i}L_{i}\left(t\right)-\theta I_{Ai}\left(t\right)\\ L_{Pi}\left(t+1\right)=L_{Pi}\left(t\right)+\sigma \left(1-\theta \right)\left(1-p_{i}\right)L_{i}\left(t\right)-\theta L_{Pi}\left(t\right)\\ -\sigma_{pre}\left(1-\theta \right)L_{Pi}\left(t\right)\\ I_{Si}\left(t+1\right)=I_{Si}\left(t\right)+\sigma_{pre}\left(1-\theta \right)L_{Pi}\left(t\right)-\theta I_{Si}\left(t\right) \end{array}$$

In this study, σ is the rate from *L_i_* to *L*_P*i*_ or *I*_A*i*_. $\sigma_{\mathrm{pre}}$ is the rate from *L*_P*i*_ to *I*_S*i*_. Therefore, 1/σ is the latent period for asymptomatic cases and pre-symptomatic cases. $1/\sigma_{\mathrm{pre}}$ is the pre-symptomatic period for the symptomatic. Let β represent the transmission probability for the effective contact with infectious symptomatic individuals. We take the transmission probability for the effective contact with infectious asymptomatic individuals to be rβ and the transmission probability for the effective contact with pre-symptomatic individuals to be kβ. $\mu_{i}$ is the susceptibility to infection for age-group *i* [38]*.* $C_{ij}$ is the number of contacts between age group *i* and age group *j* per day. *G* is the number of age groups. The values and priors for the parameters in the model are listed in electronic supplementary material, table S1. The prior information for $p_{i}$ was set according to the observation in each city.

Previous studies have shown that the contacts among different age groups changed markedly before and during the Chinese lockdown [32,39]. Considering this fact, two contact matrices describing average contacts among age groups before and during lockdown (electronic supplementary material, table S2) were incorporated into the model. For each city, the baseline contact matrix is used before lockdown date and the lockdown contact matrix is used after lockdown date. Although population-level testing and contact tracing were implemented in each city, the effectiveness probably differed among cities. To account for this, we used the location-specific proportion of infection detection by contact tracing and population-level testing (represented by parameter θ in electronic supplementary material, figure S1). A pre-symptomatic stage was also included because a COVID-19 case can become infectious before developing symptoms. We have two sets of observations: the daily new asymptomatic infections and the daily new symptomatic infections given by case onset. These observations correspond to the following equations:

$$\begin{array}{c} R_{S}(t)=\sum_{i=1}^{G}R_{Si}(t),\;\text{where }R_{Si}(t)=\theta I_{Si}(t)+\theta L_{Pi}(t)+\theta (1-p_{i})L_{i}(t),\;\\ R_{A}(t)=\sum_{i=1}^{G}R_{Ai}(t),\;\text{where }R_{Ai}(t)=\theta I_{Ai}(t)+\theta p_{i}L_{i}(t).\; \end{array}$$

In the stochastic model, we assume that the daily asymptomatic infections and the daily new symptomatic infections at time *t* followed the Poisson distribution with mean of $R_{S}(t)$ and $R_{A}(t)$, respectively. We fitted $R_{S}(t)$ and $R_{A}(t)$ to the two sets of observations with 3-day rolling mean. Model fitting was performed using the Metropolis–Hastings Markov chain Monte Carlo algorithm with the Matlab (v. R2020a) toolbox mcmcstat [40] (https://github.com/mjlaine/mcmcstat). One hundred thousand iterations were set for burn-in. After that, another 100 000 iterations were performed. For Shijiazhuang and Harbin, the onset date for the first case was a few days before the date of confirmation (i.e. the date non-pharmaceutical interventions (NPIs) were launched), and θ was set to be zero until the confirmation date for the first case. The fitting results are shown in electronic supplementary material, table S3.

### 2.3. The SARS-CoV-2 case data for six cities of China

We collected data on SARS-CoV-2 infections from six cities in China (Shijiazhuang and Xingtai from Hebei province, Changchun and Tonghua from Jilin province, Harbin and Suihua from Heilongjiang province). The population size for the six cities is approximately 9.8 million, 8.0 million, 8.6 million, 2.1 million, 9.5 million and 5.2 million, respectively. To detect all ongoing infections as early as possible, the cities launched population-level PCR tests with more than 48 million tests in total (the actual number of tests should be higher than this since not all the information about the number of PCR tests for each round of population-level testing could be collected for this study); for example, Shijiazhuang performed three rounds of testing and Xingtai conducted as many as 10 rounds (electronic supplementary material, table S4).

For each city, the detailed surveillance data were collected from local Centers for Disease Control and Prevention, including the gender, age and onset date. For each patient, the clinical endpoint (asymptomatic or symptomatic) was also collected over a multi-week period.

### 2.4. The mobility data

In China, human movements were anonymously collected at the city level with mobile phone data, through location-based services (LBS) employed by the popular Baidu applications. We used relative volume of inflows movement for each city from the migration flows database (http://qianxi.baidu.com/). The study period for Shijangzhuang, Xingtai, Changchun, Tonghua, Harbin and Suihua was 23 December 2020–12 February 2021, 1 January 2021–24 January 2021, 10 January 2021–4 February 2021, 15 January 2021–9 February 2021, 6 January 2021–8 February 2021 and 9 January 2021–5 February 2021, respectively.

### 2.5. Age-dependent contact pattern

There are no specific contact matrices for the six cities in our study. To perform the analysis, the contact matrices for Wuhan, Shanghai, Changcha and Shenzhen before and after lockdown were downloaded from the studies of Zhang *et al*. [32,39]. The number in each cell of contact matrices before lockdown were averaged across four cities. There are 14 age groups in the original contact matrix. Considering that our data may be not able to support such detailed age groups, the averaged contact matrix was merged according to the age groups in our study (i.e. seven age groups). This is the baseline contact matrix. The same procedure was applied to the contact matrices after lockdown. The lockdown contact matrix was also obtained. The cities of Shijiazhuang, Xingtai and Tonghua performed lockdown intervention, while other cities did not. To facilitate the comparison among these cities, we took the date with the lowest inner-city mobility as the lockdown date (electronic supplementary material, figure S2). Before this date, the baseline contact matrix (describing the contact rate among the different age groups) was used. After this date, the lockdown contact matrix was used. The date for lockdown and the date with the lowest inner-city mobility were closed (electronic supplementary material, table S5). Here, we used the same contact matrices (i.e. the baseline contact matrix and the lockdown contact matrix; electronic supplementary material, table S2) for each city in our study by assuming similar living habits across cities in China.

## 3. Results

### 3.1. The proportion of asymptomatic infections from multiple rounds of population-level PCR tests with patient follow-up

In order to understand heterogeneity in proportions of asymptomatic infections under demographic influence, we used detailed surveillance data gathered during COVID-19 resurgences in six cities of China at the beginning of 2021 to address this question. Previous studies show that local demography may play a role in the observed geographical and age-related heterogeneity of asymptomatic proportions (electronic supplementary material, figures S3–S5 and table S6). In this study, six cities in China (Shijiazhuang and Xingtai from Hebei province, Changchun and Tonghua from Jilin province, Harbin and Suihua from Heilongjiang province) were geographically close and had similar COVID-19 resurgences temporally, while manifesting heterogeneous asymptomatic proportions. All cities launched multi-round population-level PCR testing (applied to all the individuals in a city; electronic supplementary material, table S4) combined with detailed contact tracing and surveillance, making it a quick identification of SARS-CoV-2 infection regardless of whether displaying symptoms. Contact tracing was triggered if an individual was positive by PCR test, irrespective of the symptom status. So, the primary cases (being symptomatic or asymptomatic) from contact tracing were included in the analysis.

**Figure 1.**
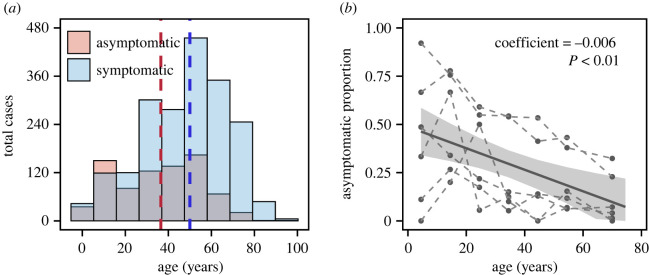
Pattern of SARS-CoV-2 infections in six cities in China before mass vaccination. (*a*) The total asymptomatic infection (light red) and symptomatic infection (light blue) distribution from six cities (Shijiazhuang, Xingtai, Changchun, Tonghua, Harbin and Suihua) in age groups. The red dashed line represents the average of age in asymptomatic SARS-CoV-2 infections. The blue dashed line represents the average of age in symptomatic SARS-CoV-2 infections. (*b*) The grey dots connected by dashed line represent asymptomatic proportion from six Chinese cities in our study. A linear regression was built (asymptomatic proportion = intercept + coefficient × age). The grey line represents the linear regression using pooled asymptomatic proportion under each age group across cities with 95% CI in light grey shadow (coefficient = −0.006, *p* < 0.01).

**Figure 2.**
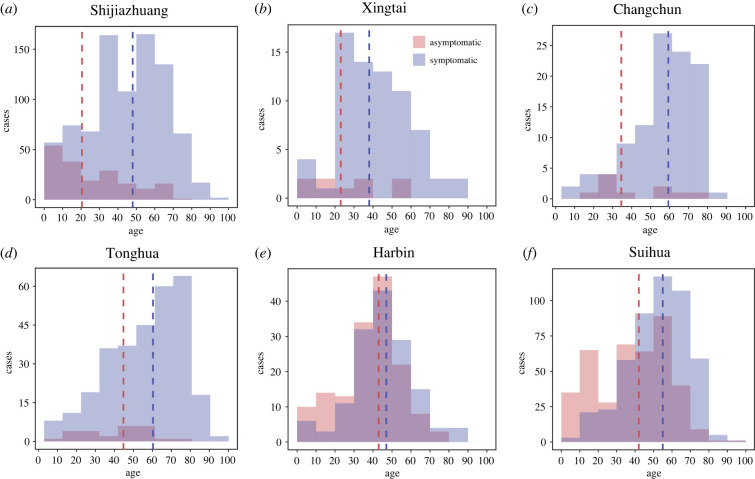
The case distribution in age groups for symptomatic infection and asymptomatic infection. (*a*) Shijiazhuang, (*b*) Xingtai, (*c*) Changchun, (*d*) Tonghua, (*e*) Harbin and (*f*) Suihua. Symptomatic infection: light purple. Asymptomatic infection: light pink. The red dashed line represents the average of age in asymptomatic infections. The blue dashed line represents the average of age in symptomatic infections.

With detailed surveillance data including information on age distribution and clinical endpoint of each infection, we first looked into the statistical property of proportion of asymptomatic SARS-CoV-2 infection among different age groups. In total, there were 2744 PCR-confirmed SARS-CoV-2 infections with the highest number of 1040 in Shijiazhuang and the lowest number of 80 in Xingtai (electronic supplementary material, table S4). Among all SARS-CoV-2 infections, there were 1964 symptomatic infections with a median age of 50 and 780 asymptomatic infections with a median age of 36, indicating younger age groups are at risk of developing infectious but asymptomatic (figure 1*a*). The age distribution of infections varied by location: the largest proportion of positive cases was in individuals aged greater than 60 in Shijiazhuang, Changchun, Tonghua and Suihua, while the largest proportion was in age group of 20–29 for Xingtai and in age group of 40–49 for Harbin (figure 2).

**Figure 3.**
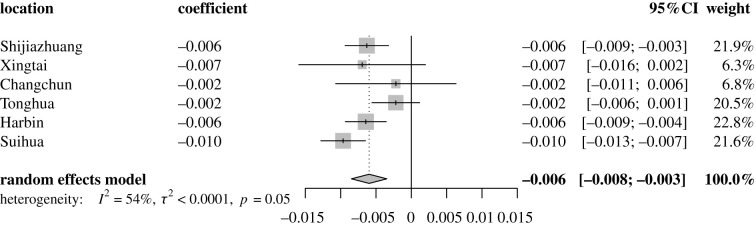
Pooled coefficient of age on the asymptomatic proportion in six Chinese cities. The coefficient was obtained from linear regression (asymptomatic proportion = intercept + coefficient × age) built for each city in our study in figure 1*b*.

**Figure 4.**
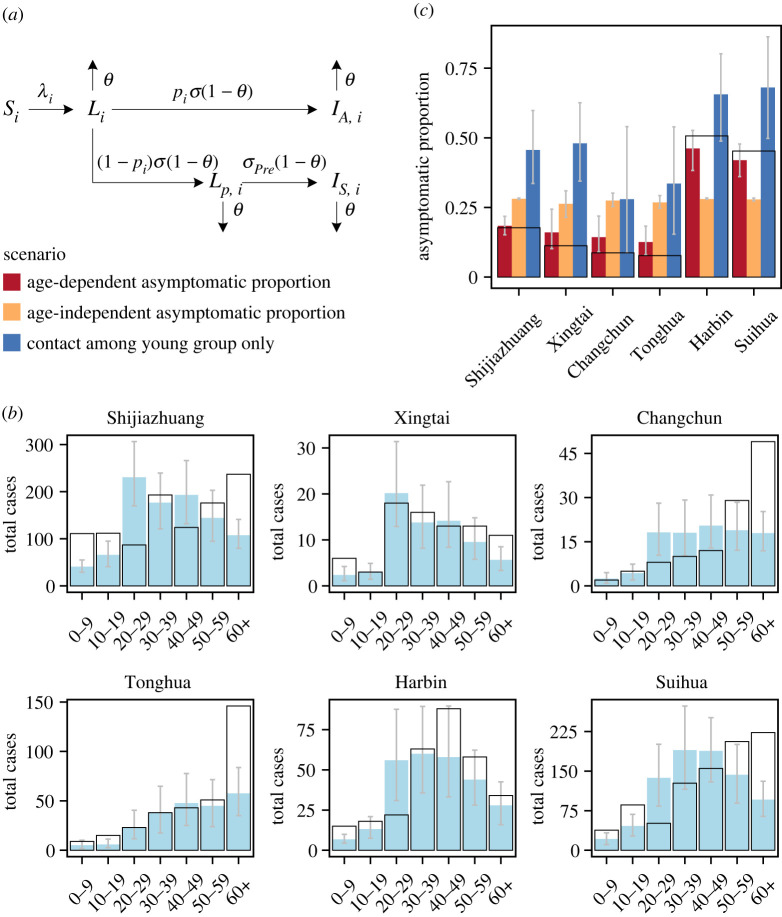
The estimated SARS-CoV-2 cases distribution in age groups in six cities and the total asymptomatic proportions under different scenarios. (*a*) Model diagram. The following compartments are considered: susceptible (*S_i_*), latent state (*L_i_*), pre-symptomatic (*L_p, i_*), infectious asymptomatic (*I_A, i_*), infectious symptomatic (*I_S, i_*) in age group *i*. *λ_i_* is the force of infection in age group *i*; 1/*σ* is the latent period for asymptomatic and pre-symptomatic; *p_i_* is the proportion of infections that manifest as asymptomatic cases in age group *i*; 1/*σ*_pre_ is the pre-symptomatic period for symptomatic; *θ* is the proportion of infection detected by contact tracing and population-level testing (see Methods). Population-level testing is applied to the individuals in a city, i.e. the individuals in all the compartments. (*b*) Estimate of the developed model to the total number of SARS-CoV-2 cases in age groups for six cities. The total number of SARS-CoV-2 cases reported (black box) and the estimated total number of cases (light blue box) in each city and each age group. The grey line represents 95% CI. (*c*) The total asymptomatic proportions under different scenarios. Red: simulated, based on the observed age-dependent asymptomatic proportions. Yellow: simulated, with age-independent asymptomatic proportions. This proportion was calculated by the total asymptomatic cases divided by the total SARS-CoV-2 cases using the pooled data from six cities. Blue: simulated, with contacts among the 0–9 and 10–19 age groups only. The grey bar represents the 95% CI for each simulation. White bars with black borders represent the observed total asymptomatic proportion in each city.

Across the six cities, the proportion of asymptomatic infections declined with age (figure 1*b*; coefficient = −0.006 (95% CI: −0.008 to −0.003) and *p* < 0.01 from linear regression). To consider the heterogenicity in each city, a meta-analysis was applied to the coefficients of age from each city. The asymptomatic proportion was still significantly associated with age (figure 3). The total or ‘crude' asymptomatic proportion (i.e. the asymptomatic SARS-CoV-2 infections divided by all the SARS-CoV-2 infections for each city; see Methods) varied considerably, from 8% in Tonghua to 18% in Shijiazhuang and 51% in Harbin (white bars with black borders in figure 4*b*). The bias in this asymptomatic proportion calculation should be very small for the following reasons: (i) the populations were tested multiple times during the COVID-19 flare-ups and (ii) the identified cases were isolated and under 14-day health monitoring to create a definite clinical outcome (asymptomatic or symptomatic) per individual. For each age group, there was heterogeneity in the probability of being asymptomatic across the cities (figure 1*b*). Identification of the underlying biological mechanism of this heterogeneity is beyond the scope of the current study, as it mainly focuses on the overall, cross-city asymptomatic proportion across all ages.

### 3.2. Age-dependent asymptomatic proportions and age distribution of cases contribute to the total asymptomatic proportion

To explore the mechanism underlying heterogeneity in overall, cross-city asymptomatic proportions, we developed an age-stratified compartment model that incorporated age-dependent asymptomatic proportions to reconstruct observed epidemic trajectories, including both asymptomatic and symptomatic cases (figure 4*a*; electronic supplementary material, figure S1). We hypothesized that cities report different total asymptomatic proportions of SARS-CoV-2 infections because of the interplay between the age-dependent asymptomatic proportion of cases and the age distribution of infections. The proportion of asymptomatic infections was assumed to decline with age, in accordance to our findings and other reports [38]. The model accurately described the daily numbers of asymptomatic and symptomatic cases (electronic supplementary material, figure S6), the case distribution by age (figure 4*b*), the age-dependent proportion of asymptomatic cases (electronic supplementary material, figure S7), and the reported asymptomatic proportion of all cases (red bar in figure 4*c*) across all the cities (note that these were not involved in the loss function during fitting).

Based on the fit of the model, we next investigated the effect of the age-dependent asymptomatic proportions on total asymptomatic proportions. Our model suggested that if the proportion of asymptomatic cases had been the same across all age groups (i.e. age-independent), the total proportion of asymptomatic cases in each city would have been equal (yellow bar in figure 4*c*; electronic supplementary material, figure S8). However, this contradicted the observations and illustrated that the asymptomatic proportion depends on age, verifying that age-dependent asymptomatic proportions contribute to the total asymptomatic proportion.

Age distribution of cases might also have effects on the proportion of asymptomatic cases, and it will be affected by the interaction of initial infection seed among age groups, age structure and contact patterns of a city. We tested different combination of these factors by setting them to be either age-specific or homogeneous in age, but it had little effect on the total asymptomatic proportion (electronic supplementary material, figure S9). Then we considered a more extreme scenario where sustained transmission occurred only among the young population (contacts assumed only to take place among age groups (0–9) and (10–19)). In this case, the total asymptomatic proportion was predicted to be higher than observed (blue bar in figure 4*b*; electronic supplementary material, figure S10), because of the shifted age distribution of cases (electronic supplementary material, figure S11*a*–*d*). These results illustrate that transmission within specific age groups, such as school transmission and nursing home transmission, will greatly alter the total asymptomatic proportion.

### 3.3. The sensitivity analysis for the six cities in China

As the susceptibility to SARS-CoV-2 infection is expected to increase with age [38], we also investigated the effect of susceptibility to infection on the total asymptomatic proportion. By the simulation with homogeneous susceptibility among different age groups, we found that the total asymptomatic proportion was higher than the observations. This is due to more cases happening among the younger groups (electronic supplementary material, figure S11*e*,*f*). We also replaced the contact matrix with the contact matrices from Wuhan, Shanghai, Changsha and Shenzhen in China [32,39] and the total asymptomatic proportion did not show dramatic changes (electronic supplementary material, figure S12). The age-dependent seed, age-structure and social contact matrices were also evaluated. Multiple simulations were performed by changing the age-dependent seed, age-structure and social contact matrices of the population to be homogeneous in age groups. The results showed that the predicted total asymptomatic proportion may be relatively unchanged among these combinations of models (electronic supplementary material, figures S13–S20).

To illustrate the robustness of our model, we tested the effects of different initial values for parameters and the estimations were robust to the different initial values (electronic supplementary material, table S7). Considering other NPIs implemented, we used the inner-city mobility as a proxy to test whether the fitting was improved or not by assuming that the reduction in mobility has an effect on the transmission probability. Specifically, transmission probability (*β*) is scaled by β*Mobility/max_Mobility, where max_Mobility is the maximum value of mobility during the outbreak. However, the model performance showed limited improvement by adding human mobility information (electronic supplementary material, table S8).

## 4. Discussion

Our study provides insight into the observed variability in the proportion of asymptomatic SARS-CoV-2 infections. Based on previous studies [6,26,41–43] and on data from the present study before mass vaccination, the proportion of cases that is asymptomatic declines with age. Together, local age-dependent asymptomatic proportions along with the age distribution of cases lead to differences in the asymptomatic proportions observed in different populations. Cities with younger populations are more likely to have a high proportion of asymptomatic infections and are at a high risk of COVID-19 endemic due to undetected asymptomatic transmission.

A great deal of concern has been directed toward the asymptomatic proportion among infected vaccinated people, which has been reported to be higher than that among the unvaccinated. However, vaccines could not have contributed to heterogeneity in the asymptomatic proportion in the six cities of our study because the vaccine roll-out had not started at the time of data collection. It is also unlikely that exposure during the first COVID-19 wave contributed to the heterogeneity of asymptomatic proportions in this dataset because the total number of SARS-CoV-2 infections during the first wave in 2020 among these six cities was small: ranging from 7 in Tonghua to 198 in Harbin. Additionally, a retrospective serological survey conducted in six provinces or municipalities (of Hubei province) from 10 April 2020 to 18 April 2020 suggested that less than 0.1% of the population carried antibodies against SARS-CoV-2 [44].

There are a few limitations in our study. Due to the lack of city-specific contact matrices, we used averaged contact matrices from four other cities in China to represent contact patterns in our research assuming similar living habits across cities in China. The sensitivity analyses showed very similar results using city-specific contact matrices. Although some studies have reported that COVID-19 vaccines lower the risk of developing symptomatic disease [45–47], we did not consider this effect of vaccines. Few studies have reported proportions of asymptomatic infections by age group or otherwise following mass vaccination campaigns. In addition, our conclusion about the heterogeneity of asymptomatic proportion may persist once vaccinated, since the effectiveness of vaccines also depends on the age. Though active surveillance was conducted in six cities and most of the infected individuals were detected by population-level testing and contact tracing, there is heterogeneity in the proportion of asymptomatic SARS-CoV-2 infection within each age group across the cities. The underlying mechanism could not be unraveled based on the current dataset, although it may be caused by small sample sizes, other biological variations in the infected population, natural variation in clinical manifestations for SARS-CoV-2 infections or testing procedures in practice between the six cities (despite standard protocols being used in all six cities). More studies would be warranted to investigate this question in the future.

In our study, most infections were identified in local communities and isolation centres. Frequent, multiple rounds of testing and contact tracing accelerated the early detection of cases and even before they showed symptoms. Routine PCR tests were also used for COVID-19 surveillance among high-risk groups (e.g. health workers) and there were no reported SARS-CoV-2 infections before the first documented cases for each city of our study. By all these means, we were able to identify asymptomatic cases. Nevertheless, the chance of finding asymptomatic and symptomatic cases could have been different. Even if this is true, it would not change our conclusion that age is a key determinant of the asymptomatic proportion. Analysing the SARS-CoV-2 infections from population-level testing would be a way to demonstrate the robustness of asymptomatic proportion calculation. However, our dataset is not a line-list (a separate line for each case) and how each individual infection was identified was unknown. This is a limitation of data collection in our study.

In summary, our results give a comprehensive profile of asymptomatic infection in six cities of China and explain factors that contribute to heterogeneity in the asymptomatic proportion. Our study provides some insights for policy related asymptomatic infection surveillance and control of future resurgences by taking age structure into consideration, especially in the era of vaccination and variants such as the Omicron era.

## Data Availability

All datasets are publicly available. This work is licensed under a Creative Commons Attribution 4.0 International (CC BY 4.0) licence, which permits unrestricted use, distribution and reproduction in any medium, provided the original work is properly cited. Code and data are available at the following repository: https://github.com/WWWMaggie/COVID-19_Role_Of_Age. The data are provided in electronic supplementary material [48].
